# Medial open transversus abdominis plane (MOTAP) catheters for analgesia following open liver resection: study protocol for a randomized controlled trial

**DOI:** 10.1186/1745-6215-15-241

**Published:** 2014-06-21

**Authors:** Paul Karanicolas, Sean Cleary, Paul McHardy, Stuart McCluskey, Jason Sawyer, Salima Ladak, Calvin Law, Alice Wei, Natalie Coburn, Raynauld Ko, Joel Katz, Alex Kiss, James Khan, Srinivas Coimbatore, Jenny Lam-McCulloch, Hance Clarke

**Affiliations:** 1 Department of Surgery, Sunnybrook Health Sciences Centre, Toronto, Canada; 2 Department of Surgery, University of Toronto, Toronto, Canada; 3 Department of Surgery, University Health Network, Toronto, Canada; 4 Department of Anaesthesia, Sunnybrook Health Sciences Centre, Toronto, Canada; 5 Department of Anaesthesia, University of Toronto, Toronto, Canada; 6 Department of Anaesthesia and Pain Management, University Health Network, Toronto, Canada; 7 Clinical Epidemiology Unit, Sunnybrook Research Institute, Toronto, Canada

**Keywords:** Liver surgery, Analgesia, Transversus abdominis plane, Randomized controlled trial

## Abstract

**Background:**

The current standard for pain control following liver surgery is intravenous, patient-controlled analgesia (IV PCA) or epidural analgesia. We have developed a modification of a regional technique called medial open transversus abdominis plane (MOTAP) catheter analgesia. The MOTAP technique involves surgically placed catheters through the open surgical site into a plane between the internal oblique muscle and the transverse abdominis muscle superiorly. The objective of this trial is to assess the efficacy of this technique.

**Methods/design:**

This protocol describes a multicentre, prospective, blinded, randomized controlled trial. One hundred and twenty patients scheduled for open liver resection through a subcostal incision will be enrolled. All patients will have two MOTAP catheters placed at the conclusion of surgery. Patients will be randomized to one of two parallel groups: experimental (local anaesthetic through MOTAP catheters) or placebo (normal saline through MOTAP catheters). Both groups will also receive IV PCA. The primary endpoint is mean cumulative postoperative opioid consumption over the first 2 postoperative days (48 hours). Secondary outcomes include pain intensity, patient functional outcomes, and the incidence of complications.

**Discussion:**

This trial has been approved by the ethics boards at participating centres and is currently enrolling patients. Data collection will be completed by the end of 2014 with analysis mid-2015 and publication by the end of 2015.

**Trial registration:**

The study is registered with http://clinicaltrials.gov ( NCT01960049; 23 September 2013)

## Background

### Background and rationale

Liver resection is the optimal treatment for patients with primary or metastatic liver malignancies, benign liver tumours, and some biliary diseases [1-5]. In Canada, over 2,000 patients annually undergo liver resection, predominantly for cancer [6]. Over the past few decades, improvements in surgical technique, better haemostatic control, and wider indications for liver resection, especially for those with metastatic disease, has resulted in a significant increase in the prevalence of liver resection surgery. There has been an increase of approximately 12% in the number of liver resections performed each year with a cumulative increase over 5 years of more than 60% [7].

Due to the location of the liver and the propensity for bleeding during resection, surgery is typically performed through a large right subcostal incision. The subcostal incision, however, is associated with significant postoperative pain and distress which may result in respiratory complications, delayed mobilization and physiotherapy, and prolonged hospital stay [8]. Inadequate postoperative pain management is associated with the development of chronic postsurgical pain which is costly to both the patient and society [9-12]. Intense postoperative pain is a risk factor for the development of chronic postsurgical pain (CPSP) [9-12]. Prolonged pain after surgery is the result of changes in the peripheral and central nervous system causing amplification, increased sensitivity, and prolongation of pain [13]. It is estimated that as many as 30% of Canadians who undergo an operation will develop CPSP and the severity of acute postoperative pain is a predictor of its development [14]. Chronic pain is costly to the patient and society in terms of direct health care expenditures, and indirect costs associated with lost productivity, as well as suffering and disability [12]. Aggressive and effective early postoperative pain relief may prevent the development of chronic pain [11].

Conventional techniques of postoperative analgesia include epidural catheters, intravenous opioid patient-controlled analgesia, and peripheral nerve blocks [15-17]. Peripheral nerve blocks - specifically transversus abdominis plane (TAP) blocks - have been described [18-20]. A modification of the TAP technique which involves surgeon placement of the TAP catheters directly into the open surgical wound (medial open transversus abdominis plane; MOTAP) has been investigated both at Sunnybrook Health Sciences Centre and at the University Health Network – Toronto General Division [21].

### Epidural analgesia

Epidural analgesia remains the gold standard for pain control following an abdominal surgical procedure [22]. However, complications from epidural analgesia include hypotension, bradycardia dural puncture, spinal infection, and rarely, but devastatingly, neurological damage [23-26]. Furthermore, removal of epidural catheters may provide logistic challenges if patients develop coagulopathy on the basis of liver dysfunction [27-30]. Given the above risks, many institutions do not routinely insert epidurals for patients undergoing major liver resection.

### Intravenous opioid patient-controlled analgesia

Despite the variety of pain-relieving modalities available, postoperative pain following liver surgery is significant and remains a challenge to manage. Opioid analgesia, while effective at rest, fails to provide adequate pain relief associated with movement such as breathing, coughing, ambulation, and gastrointestinal motility [11,31]. Moreover, opioids are associated with adverse effects such as nausea, vomiting, constipation, sedation, and respiratory depression [20].

### Transversus abdominis plane

Movement-evoked incisional pain accounts for the majority of pain experienced, and the nerves responsible arise from thoracic levels T6 to T10 [32]. These nerves lie in a plane between the internal oblique and transversus abdominis muscles, known as the TAP [18,33,34]. The TAP block involves injecting local anaesthetics into this fascial plane, thereby blocking transmission of the sensory neurons responsible for abdominal surgical pain. Afferent sensory information from these nerves can be blocked by infiltrating local anaesthetic via a surgically placed catheter that runs in the lateral TAP (blocking T8 to T10) and extends into a second adjacent anatomical compartment, the posterior aspect of the rectus sheath (blocking T6 to T8). A variation of the TAP block, the oblique subcostal block, provides sensory blockade for surgical incisions between T6 and T10 dermatomes, including the subcostal or chevron incision used in liver surgery [18]. A retrospective study of 36 patients demonstrated that ultrasound-guided TAP blocks significantly decreased 24-hour cumulative morphine consumption [19]. A recent randomized controlled trial demonstrated that TAP blocks and epidural analgesia are equally efficacious in controlling postoperative pain [20]. However, due to the close proximity to the peritoneum and the highly vascularized liver, single-shot subcostal TAP blocks are quite challenging to perform, even under ultrasound guidance, as evidenced by two cases of liver puncture in a sample of 36 patients [19]. In small studies, TAP blocks decreased postoperative opioid consumption, nausea, and vomiting, while providing pain control similar to epidural analgesia [35]. TAP blocks are typically infiltrated using ultrasound guidance at the conclusion of the operation. Preliminary uncontrolled studies and case reports have demonstrated the potential efficacy, safety, and patient satisfaction of TAP catheters [20,36].

### MOTAP technique – modification of the transversus abdominis plane technique

We have recently developed a modification of the TAP technique for regional analgesia following subcostal incisions termed MOTAP catheter placement. This technique involves surgical placement of the catheter under direct vision. During subcostal incision these muscular planes are clearly visible, allowing more confident insertion of a catheter directly into the correct plane [37]. In a pilot study, 22 fit adults undergoing donor hepatectomies received TAP catheters at the end of surgery. The patients received the first dose of bupivacaine (0.2 cm^3^/kg 0.125%) at the end of surgery and this regimen was repeated every 12 hours until postoperative day 3. We compared postoperative pain scores and opioid use to historical controls and found a significant (*P* < 0.05) difference in cumulative postoperative opioid (hydromorphone) consumption at 48 hours (mean ± SD: 20.8 ± 15.8 mg for MOTAP catheters vs 39.1 ± 25.1 mg for patient-controlled analgesia (PCA)).

### Primary objective

To assess the impact of MOTAP catheters plus intravenous, patient-controlled analgesia (IV PCA) compared to the standard of care (that is, IV PCA alone) on opioid consumption in patients undergoing hepatic resection requiring a subcostal incision.

### Secondary objectives

To assess the effect of MOTAP catheters on:

1. Postoperative pain intensity.

2. Opioid-related adverse effects, including nausea, vomiting, pruritus, sedation, and postoperative ileus.

3. Time to ambulation, duration of hospital stay and reduced postoperative pain disability.

4. Post-surgical complications (for example, pneumonia).

5. Incidence and intensity of chronic postsurgical pain.

## Methods/design

### Study design

This is a multicentre, prospective, blinded, randomized controlled trial comparing MOTAP catheters plus IV PCA to standard care (IV PCA alone) in patients undergoing subcostal incision for liver resection. Full inclusion and exclusion criteria for the trial are summarized in Table 1. The trial has been approved by the Toronto Academic Health Sciences Network Research Ethics Boards from Sunnybrook Health Sciences Centre (178–2013) and from the University Health Network (12-0493-A).

**Table 1:** MOTAP study inclusion and exclusion criteria

**Inclusion criteria**	**Exclusion criteria**
• >18 years of age	• Patients unable to comprehend instructions, consent, or co-operate with pain assessment
• Undergoing liver resection using a subcostal incision (upper midline extension is allowed)	
• Incision extends to allow visualization of internal oblique and transversus abdominis muscles	• Allergy to any study medications
	• Patient not able to be extubated postoperatively for any clinical reason
	• Laparoscopic surgery
	• Co-existing epidural or intrathecal analgesia
	• Chronic pain disorders or on long-term opioids (greater than 1 month prior to operation)
	• History of substance or alcohol abuse
	• Transplant donor liver resections
	Prior right subcostal incision

MOTAP, medial open transversus abdominis plane.

### Recruitment

Patients meeting the eligibility criteria will be recruited at the preoperative surgical consent visit or the anaesthesia pre-assessment clinic. Information pamphlets will be provided and written informed consent will be obtained. At the time of enrolment, patients will complete baseline questionnaires: the ‘Preoperative Current Pain and Pain History Questionnaire’, Numeric Rating Scale (NRS) [30] for Pain Intensity and Pain Unpleasantness, the Pain Catastrophizing Scale (PCS) [31], the McGill Pain Questionnaire - Short Form-2 (SF-MPQ-2) [32,33], and the Hospital Anxiety and Depression Scale (HADS) [34].

### Randomization

**Figure 1:**
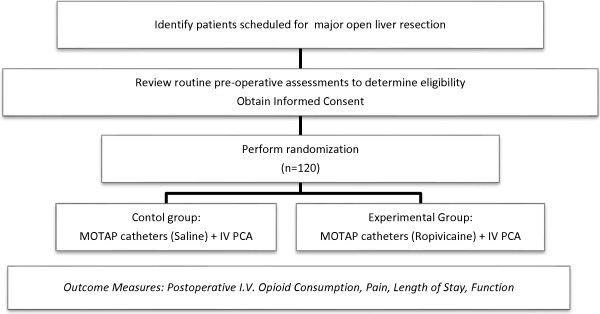
**MOTAP study schema and study outline.** IV, intravenous; MOTAP, medial open transversus abdominis plane; PCA, patient-controlled analgesia.

Patients will be randomized to one of two parallel groups: experimental (local anaesthetic through MOTAP catheter) or placebo (normal saline through MOTAP catheter). Each research pharmacy will have a concealed randomization list and the appropriate syringes will be dispensed by the pharmacy. The code will be retained centrally and only revealed to the investigators once recruitment and data collection is completed. Randomization will be stratified by centre. A brief schema and study outline of this trial is summarized in Figure 1.

### Interventions

#### Preoperative care (both groups)

All patients will undergo routine preoperative testing at the preadmission clinic to confirm fitness for surgery (medical history, electrocardiogram, blood work, and so forth). No additional tests are required beyond standard-of-care for patients undergoing liver resection.

#### Intraoperative care (both groups)

Patients will undergo routine anaesthesia and liver surgery with no additional requirements for this study. No preoperative oral analgesics are permitted, including non-steroidal anti-inflammatory drugs, gabapentin, and acetaminophen. Epidural catheters, intravenous lidocaine, and ketamine are not permitted. Surgery will be performed through a right subcostal incision. An upper midline extension or limited left subcostal extension is permitted. All aspects of the liver resection will be left to the surgeon’s discretion. Local anaesthetic may not be infiltrated into the surgical wound.

#### Insertion of MOTAP catheters (both groups)

Following liver resection, the operating surgeon will insert two catheters. The following muscles will be identified: rectus abdominis, transversus abdominis, internal oblique, and external oblique. The surgeon will dissect between the internal oblique and transversus abdominis fascia superiorly to open the TAP. A plane will also be developed between the rectus muscle and the posterior sheath. The first catheter will be placed subcostally in the anterior axillary line, between the transversus and internal abdominal muscles. The second catheter will be sited at the lateral border of the right rectus abdominis muscle, between the posterior rectus sheath and the rectus abdominis muscle. Approximately 10 cm of catheter will be left in each plane, and tunnelled to skin sites distal to insertion. The catheters will be secured under occlusive sterile dressing, and covered with tape. The skin will be closed using either staples or suture at the surgeon’s discretion. The catheter will be fastened to the skin using steri-strips and an occlusive sterile dressing. For the TAP catheter we will use the 001159-20D InfiltraLong 600 T (manufacturer Pajunk, Germany) and we will use a standard three-orifice catheter for the rectus space.

The study lead at each site will be responsible for ensuring that all participating surgeons adhere to this technique of MOTAP insertion. Prior to enrolling a patient, each participating surgeon must perform at least one MOTAP insertion with the site’s lead present to become credentialed in the technique. We will audit the insertion technique of each surgeon at least every five cases to ensure consistency.

#### Experimental group - local anaesthetic infusion through MOTAP

At the conclusion of surgery, both catheters will be bolused with 20 ml ropivacaine 0.2% followed by a continuous infusion through each catheter of ropivacaine 0.2% 5 ml per hour.

#### Control group – normal saline infusion through MOTAP

At the conclusion of surgery, patients in the control group will have 0.9% normal saline 20 ml bolused then a 5 ml/hour infusion into each catheter.

#### Postoperative care (both groups)

Patients in both groups will be prescribed celecoxib 200 mg orally twice daily (if baseline serum creatinine is less than 90 μmol/l), and IV PCA hydromorphone 0.2 mg bolus with a lockout time of 5 minutes and no background infusion. If patients have inadequate analgesia with this regimen the bolus dose will be increased to 0.2 to 0.4 mg bolus and the TAP catheter infusions will be increased to 7.5 ml/hour in each catheter. If pain is still inadequately controlled, patients will be started on a continuous intravenous infusion of hydromorphone 0.2 mg/hour in addition to the bolus PCA dosing. If pain is still inadequately controlled, the study coordinator will be contacted and the patient assessed by the study site lead.

Patients may receive additional opioid boluses to control acute intractable pain while the IV PCA dose is being adjusted, at the discretion of the acute pain service clinician or the post-anaesthesia care unit nurse caring for the patient. These doses will be recorded and included in the measurement of total opioid consumption.

Serum creatinine will be monitored daily in the postoperative period and, if it increases above 90 μmol/l, the celecoxib will be discontinued; however, patients will remain in the trial.

The MOTAP catheters will be removed on the morning of postoperative day 3 in all patients.

### Blinding

This will be a blinded, placebo-controlled study. All patients, clinicians, and data collectors will be unaware of the group allocation. A pharmacist not involved in patient care will dispense the study medications according to the computer-generated randomization list.

### Outcome assessment

#### Primary outcome

The primary endpoint is mean cumulative postoperative opioid consumption over the first 2 postoperative days (48 hours). Both IV PCA opioids and oral opioids will be recorded from the patient’s medical records daily, converted to morphine equivalents, and compared between the two groups.

The following information will be collected at regular intervals as a measure of the primary endpoints: cumulative IV PCA opioid consumption every 12 hours for 72 hours as well as total opioid consumption for the entire hospital stay.

#### Secondary outcomes

We will collect the following secondary patient-reported outcome scores:

1. NRS [30] scores for pain at rest and with coughing (0 = no pain, 10 = worst possible pain) three times daily (TID) for 48 hours and then daily until discharge.

2. Preoperative Current Pain and Pain History Questionnaire, NRS for Pain Intensity and Pain Unpleasantness, PCS [31], SF-MPQ-2 [32,33], Pain Disability Index (PDI) [36,37], and HADS [34] at baseline.

3. NRS patient satisfaction scores regarding pain control (0 = totally satisfied, 10 = totally dissatisfied) TID for the first 48 hours and then daily until discharge.

4. NRS for Pain Intensity and Pain Unpleasantness, PCS [31], SF-MPQ-2 [32,33], and HADS [34] on the day of discharge.

5. Long-term pain Follow-Up Pain Questionnaire, PDI [36,37], PCS [31], SF-MPQ-2 [32,33], HADS [34], and The Follow-Up Questionnaire 6 months following surgery (via a routine follow-up telephone call).

6. In-hospital opioid-related side effects - presence of nausea, vomiting, pruritus, and dizziness (assessed TID for the first 48 hours, then daily until discharge).

7. Sedation scores, measured TID for the first 48 hours, then daily until discharge using a standard sedation scale (0 = alert, 1 = mild (occasionally drowsy, easy to arouse), 2 = moderate (frequently drowsy, easy to arouse), 3 = severe (somnolent, difficult to arouse), S = normal sleep, easy to arouse).

8. Duration of hospital stay.

9. Time to first bowel movement.

10. Time to ambulation.

11. Incidence of in-hospital complications, graded using the Clavien classification [38].

### Criteria for removal from study

If, at any time, the patient is found to be ineligible for the protocol as designated in the section on eligibility the patient will be removed from the study. Patients who are not extubated within 4 hours from the end of the operation or who are reintubated within 48 hours from the end of the operation (including a return to the operating room) will also be excluded from the primary analysis, although we will continue to follow them while in hospital. Patients who wish to withdraw from the study at any point in time will be able to do so.

### Data analysis

All statistical analyses will be performed using SAS software version 9.2 (SAS Inc., Cary, NC, USA). All tests will be two-sided and significance will be considered at *P* < 0.05.

#### Primary analysis

Mean cumulative postoperative opioid consumption during the first postoperative 48 hours will be reported for the two groups as mean ± standard deviation. The two groups will be compared using a two sided *t*-test.

#### Secondary outcomes

Outcomes will be reported for the two groups as mean ± standard deviation for normally distributed variables and the median and interquartile range for nonparametric variables. The two groups will be compared using chi-square tests, two sample Student’s *t*-test or the Wilcoxon rank-sum test as appropriate. Time-to-event outcomes will be defined from the completion of surgery until the time of event. Patients who do not have the event at last follow-up will be censored. The number of censored events is expected to be low as both events (ambulation and bowel movements) are requirements prior to the patient’s discharge from hospital. Time to event outcomes will be analyzed using the Kaplan-Meier method and survival curves will be compared between the two groups using the log-rank test.Sample size calculation

Our pilot data consisted of 111 patients that had undergone liver resection surgery between April 2004 and January 2009 prior to the use of MOTAP catheters when postoperative pain relief was provided by IV PCA hydromorphone alone. Cumulative hydromorphone consumption over the first 48 hours after surgery was 39.09 ± 25.07 mg. Based on our experience with the use of MOTAP catheters following adult liver donation surgery, we have found a reduction of 47% in mean cumulative opioid consumption over the first 2 postoperative days (48 hours). Patients receiving MOTAP catheters consumed a mean ± SD of 20.81 ± 15.8 mg of hydromorphone. The difference of approximately 18 mg over 48 hours is clinically significant and represents a reduction of almost 50%.

To be conservative, we will estimate a relative reduction in mean cumulative opioid consumption of 40%. Assuming a baseline opioid consumption of 39.09 mg with a standard deviation of 25.07, a sample size of 58 patients per group will achieve 90% power at a significance level (alpha) of 0.05. We will round up to 120 patients in total (60 patients in each group).

## Discussion

Data from this study will help us optimize postoperative pain management in patients undergoing liver resection. It will assist us in developing a standardized protocol for pain management for these operations. Based on the results of this work, we hope to further test the efficacy of these catheters in other surgical cohorts in which upper abdominal incisions are used and where central neuraxial blocks are either contraindicated or refused by the patient. Better control of pain will help improve patient’s pain experience, mobilization and physiotherapy following surgery, reduce pain disability and possibly decrease the incidence and intensity of CPSP.

## Trial status

This trial has been approved by the ethics boards at participating centres and is currently enrolling patients. Data collection will be completed by the end of 2014 with analysis mid-2015 and publication by the end of 2015.

### Ethics approval

Toronto Academic Health Sciences Network Research Ethics Boards from Sunnybrook Health Sciences Centre and from the University Health Network.

## Abbreviations

CPSP: chronic postsurgical pain; HADS: Hospital Anxiety and Depression Scale; IV PCA: intravenous, patient-controlled analgesia; MOTAP: medial open transversus abdominis plane; NRS: Numeric Rating Scale; PCA: patient-controlled analgesia; PCS: Pain Catastrophizing Scale; PDI: Pain Disability Index; SF-MPQ-2: McGill Pain Questionnaire - Short Form-2; TAP: transversus abdominis plane; TID: three times daily.

## Competing interests

The authors declare that they have no competing interests.

## Authors’ contributions

PK conceived the study, participated in the design, coordination, and data collection of the study, and wrote the first draft of the manuscript. SC and PM participated in the design, coordination, and data collection of the study, and revised the manuscript. SM, JS, SL, CL, AW, NC, RK, JKa, SC, and JLM participated in the design and data collection of the study, and revised the manuscript. AK participated in the design and statistical analysis of the study, and revised the manuscript. JKh participated in the design of the study, and revised the manuscript. HC conceived the study, participated in the design, coordination, and data collection of the study, and revised the manuscript. All authors read and approved of the final version of the manuscript.

## References

[B1] Beard S Holmes H Price C Majeed AW Hepatic resection for colorectal liver metastasis: a cost-effectiveness analysis Ann Surg 2000 232 763 776 11088071 10.1097/00000658-200012000-00005 PMC1421269

[B2] Lin T Lee C Chen KM Chen CC Role of surgery in the treatment of primary carcinoma of the liver: a 31-year experience Br J Surg 1987 74 839 842 2822201 10.1002/bjs.1800740931

[B3] Man K Fan ST Ng IO Lo CM Liu CL Wong J Prospective evaluation of Pringle maneuver in hepatectomy for liver tumors by a randomized study Ann Surg 1997 226 704 711 9409569 10.1097/00000658-199712000-00007 PMC1191142

[B4] Poon RT Fan ST Lo CM Liu CL Lam CM Yuen WK Yeung C Wong J Improving perioperative outcome expands the role of hepatectomy in management of benign and malignant hepatobiliary diseases: analysis of 1222 consecutive patients from a prospective database Ann Surg 2004 240 698 710 15383797 10.1097/01.sla.0000141195.66155.0c PMC1356471

[B5] Ramos E Dalmau A Sabate A Lama C Llado L Figueras J Jaurrieta E Red blood cell transfusion in liver transplantation: influence on patient outcome, prediction of requirements, and measures to reduce them Liver Transpl 2003 9 1320 1327 14625833 10.1016/jlts.2003.50204

[B6] McColl R Brar B Ghali W Dixon D Hepatic resection in Canada: rates and geographic variation Can J Surg 2009 52 E264 E268 20011162 PMC2792413

[B7] Lyratzopoulos G Tyrrell C Smith P Yelloly J Recent trends in liver resection surgery activity and population utilization rates in English regions HPB 2007 9 277 280 18345304 10.1080/13651820701504165 PMC2215396

[B8] Morrison RS Magaziner J McLaughlin MA Orosz G Silberzweig SB Koval KJ Siu AL The impact of post-operative pain on outcomes following hip fracture Pain 2003 103 303 311 12791436 10.1016/S0304-3959(02)00458-X

[B9] Katz J Pain begets pain - predictors of long-term phantom limb pain and post-thoracotomy pain Pain Forum 1997 6 140 144

[B10] Katz J Seltzer Z Transition from acute to chronic postsurgical pain: risk factors and protective factors Expert Rev Neurother 2009 9 723 744 19402781 10.1586/ern.09.20

[B11] Kehlet H Jensen T Woolf C Persistent postsurgical pain: risk factors and prevention Lancet 2006 367 1618 1625 16698416 10.1016/S0140-6736(06)68700-X

[B12] Perkins F Kehlet H Chronic pain as an outcome of surgery. A review of predictive factors Anesthesiol 2000 93 1123 1133 10.1097/00000542-200010000-00038 11020770

[B13] Fregni F Freedman S Pascual-Leone A Recent advances in the treatment of chronic pain with non-invasive brain stimulation techniques The Lancet Neurology 2007 6 188 191 17239806 10.1016/S1474-4422(07)70032-7

[B14] Goldstein DH Ellis J Brown R Wilson R Penning J Chisom K VanDenKerkhof E Recommendations for improved acute pain services: Canadian collaborative acute pain initiative Pain Res Manag 2004 9 123 130 15340582 10.1155/2004/397452

[B15] Galinski M Delhotal-Landes B Lockey DJ Rouaud J Bah S Bossard AE Lapostolle F Chauvin M Adnet F Reduction of paracetamol metabolism after hepatic resection Pharmacology 2006 77 161 165 16837779 10.1159/000094459

[B16] Rafii A Querleu D Laparoscopic obturator nerve neurolysis after pelvic lymphadenectomy J Minim Invasive Gynecol 2006 13 17 19 16431318 10.1016/j.jmig.2005.08.008

[B17] Wrighton LJ O’Bosky KR Namm JP Senthil M Postoperative management after hepatic resection J Gastrointest Oncol 2012 3 41 47 22811868 10.3978/j.issn.2078-6891.2012.003 PMC3397638

[B18] Sforza M Andjekov K Zaccheddu R Nagi R Colic M Transversus abdominis plane block anesthesia in abdominoplasties Plast Reconstr Surg 2011 128 529 535 21788846 10.1097/PRS.0b013e31821e6f51

[B19] Milan ZB Duncan B Rewari V Kocarev M Collin R Subcostal transversus abdominis plane block for postoperative analgesia in liver transplant recipients Transplant Proc 2011 43 2687 2690 21911147 10.1016/j.transproceed.2011.06.059

[B20] Niraj G Kelkar A Fox AJ Oblique sub-costal transversus abdominis plane (TAP) catheters: an alternative to epidural analgesia after upper abdominal surgery Anaesthesia 2009 64 1137 1140 19735408 10.1111/j.1365-2044.2009.06006.x

[B21] Behman R McHardy P Sawyer J Lam-McCulloch J Law C Coburn N Hanna S Karanicolas P Medial open transversus abdominis plane (MOTAP) catheter analgesia: a simple, safe, effective technique following liver resection HPB 2013 15 Suppl 1 6

[B22] Clarke H Chandy T Srinivas C Ladak S Okubo N Mitsakakis N Holtzman S Grant D McCluskey SA Katz J Epidural analgesia provides better pain management after live liver donation: a retrospective study Liver Transpl 2011 17 315 331 21384514 10.1002/lt.22221

[B23] Horlocker TT McGregor DG Matsushige DK Schroeder DR Besse JA A retrospective review of 4767 consecutive spinal anesthetics: central nervous system complications. Perioperative Outcomes Group Anesth Analg 1997 84 578 584 9052305 10.1097/00000539-199703000-00021

[B24] Kaiser AM Zollinger A De Lorenzi D Largiader F Weder W Prospective, randomized comparison of extrapleural versus epidural analgesia for postthoracotomy pain Ann Thorac Surg 1998 66 367 372 9725371 10.1016/s0003-4975(98)00448-2

[B25] Liu S Carpenter RL Neal JM Epidural anesthesia and analgesia. Their role in postoperative outcome Anesthesiol 1995 82 1474 1506 10.1097/00000542-199506000-00019 7793661

[B26] Tanaka K Watanabe R Harada T Dan K Extensive application of epidural anesthesia and analgesia in a university hospital: incidence of complications related to technique Reg Anesth 1993 18 34 38 8448096

[B27] Page A Rostad B Staley CA Levy JH Park J Goodman M Sarmiento JM Galloway J Delman KA Kooby DA Epidural analgesia in hepatic resection J Am Coll Surg 2008 206 1184 1192 18501817 10.1016/j.jamcollsurg.2007.12.041

[B28] Page AJ Kooby DA Perioperative management of hepatic resection J Gastrointest Oncol 2012 3 19 27 22811866 10.3978/j.issn.2078-6891.2012.005 PMC3397643

[B29] Schumann R Zabala L Angelis M Bonney I Tighiouart H Carr DB Altered hematologic profiles following donor right hepatectomy and implications for perioperative analgesic management Liver Transpl 2004 10 363 368 15004762 10.1002/lt.20059

[B30] Shontz R Karuparthy V Temple R Brennan TJ Prevalence and risk factors predisposing to coagulopathy in patients receiving epidural analgesia for hepatic surgery Reg Anesth Pain Med 2009 34 308 311 19574863 10.1097/AAP.0b013e3181ac7d00

[B31] Mann C Pouzeratte Y Boccara G Peccoux C Vergne C Brunat G Domergue J Millat B Colson P Comparison of intravenous or epidural patient-controlled analgesia in the elderly after major abdominal surgery Anesthesiology 2000 92 433 441 10691230 10.1097/00000542-200002000-00025

[B32] Scott N Mogensen T Greulich A Hjorso N Kehlet H No effect of continuous i.p. infusion of bupivacaine on postoperative analgesia, pulmonary function and the stress response to surgery Br J Anaesth 1988 61 165 168 3415889 10.1093/bja/61.2.165

[B33] O’Donnell B McDonnell J McShane A The transversus abdominis plane (TAP) block in open retropubic prostatectomy Reg Anesth Pain Med 2006 31 91 16418039 10.1016/j.rapm.2005.10.006

[B34] Rozen W Tran T Ashton M Barrington M Ivanusic J Taylor G Refining the course of the thoracolumbar nerves: a new understanding of the innervation of the anterior abdominal wall Clin Anat 2008 21 325 333 18428988 10.1002/ca.20621

[B35] Chan SK Lai PB Li PT Wong J Karmakar MK Lee KF Gin T The analgesic efficacy of continuous wound instillation with ropivacaine after open hepatic surgery Anaesthesia 2010 65 1180 1186 20958277 10.1111/j.1365-2044.2010.06530.x

[B36] Basu S Tamijmarane A Bulters D Wells JK John TG Rees M An alternative method of wound pain control following hepatic resection: a preliminary study HPB (Oxford) 2004 6 186 189 18333074 10.1080/13651820410030844 PMC2020673

[B37] Behman R McHardy P Sawyer J Lam-McCulloch J Karanicolas P Open transversus abdominis plane (MOTAP) catheter analgesia - a simple, safe, effective technique following open liver resection J Am Coll Surg 2014 in press 10.1016/j.jamcollsurg.2013.12.054 24745582

[B38] Clavien PA Barkun J de Oliveira ML Vauthey JN Dindo D Schulick RD de Santibañes E Pekolj J Slankamenac K Bassi C Graf R Vonlanthen R Padbury R Cameron JL Makuuchi M The Clavien-Dindo classification of surgical complications: five-year experience Ann Surg 2009 250 187 196 19638912 10.1097/SLA.0b013e3181b13ca2

